# Resistive Response of Carbon Nanotube-Based Composites Subjected to Water Aging

**DOI:** 10.3390/nano11092183

**Published:** 2021-08-25

**Authors:** Liberata Guadagno, Luigi Vertuccio

**Affiliations:** Department of Industrial Engineering, University of Salerno, Via Giovanni Paolo II, 84084 Fisciano, Italy; lguadagno@unisa.it

**Keywords:** resistive response, water sorption, water diffusion model, epoxy resin, carbon nanotubes

## Abstract

This work aimed to monitor, through the changes in electrical resistance, the evolution of the mechanical properties due to aging caused by water sorption in carbon nanotube-based epoxy composites. The epoxy/CNT nanocomposites were prepared by dispersing the filler in the precursor through the ultra-sonication process and mixing the hardener by mechanical stirring. After an evaluation of the electrical properties, detected through a two-probe electrical measurement method, of nanocomposites at different percentages by weight of the filler (0.025, 0.05, 0.1, 0.3, 0.5, and 1.0), a concentration (0.1% by weight), close to that of the electrical percolation threshold, was chosen to evaluate the resistive response. This specific concentration was selected in order to obtain maximized values of the variation detected for the changes in the electrical resistance resulting from phenomena of structural relaxations/rearrangements due to water absorption. In particular, the electrical conductivity value switched from 8.2 × 10^−14^ S/m for the unfilled epoxy resin to 6.3 × 10^−2^ S/m for carbon nanotube-based epoxy composite at 0.1% by weight of the nanofiller. The water sorption caused a reduction in the mechanical properties (storage modulus and tan δ) due to swelling and plasticization phenomena, which caused the structural reorganization of the conductive interparticle contacts in the matrix with a consequent variation in the electrical resistance of the material. The found ‘non-Fickian’ water diffusion behavior was very similar to the variation in the electrical resistance with time. This last correlation allows the association of the measurement of the electrical resistance with the quantity of absorbed water and, therefore, with the aging of the material to water absorption, through the sensitivity factor (β). The resistive nature of the composite can be used to monitor the amount of water absorption and the changes in the structure of the material subject to water aging.

## 1. Introduction

Epoxy resins are typical materials used as matrices for structural composites in aerospace, automotive, and civil engineering infrastructures [1,2,3]. Their success is due to their good thermal and mechanical performance combined with their chemical resistance. Compared to traditional materials, such as metal, metal alloys, and reinforced concrete, they manifest high values of the stiffness-to-weight and strength-to-weight ratios, together with high resistance to corrosive agents [4]. However, one of the aspects to be taken into consideration, for specific applications, is their sensitivity to the moisture content [5,6]. In many applications, the epoxy resins are exposed to a humid environment and/or high temperatures [5]. The water uptake usually causes a decrease in mechanical performance of the epoxy-based component. In particular, the penetration of water in the matrix causes an increase in mobility of the chain segments, which then causes reductions in the storage modulus, glass transition temperature, etc. [5,6,7,8]. Modifications of the structure of the polymer [5] and/or the introduction of fillers allowed the improvement of the water sorption properties [9,10,11] and to introduce additional functionalities [12,13,14,15,16,17]. The inclusion of nanosize carbon fillers usually confers an improvement to the desired properties of the hosting polymeric matrices [5,10,12,14,18]. For instance, it is known that many mechanical parameters of polymeric materials are very sensitive to photo-oxidative degradation [18]. CNTs are able to confer improved mechanical properties [5,12] and contrast the chemical degradation due to sunlight exposure [14]. Furthermore, the electrical properties of the nanofillers can be partially conferred to the matrices to contrast the electrical insulating properties of polymeric materials [10].

A more recent, innovative, and much-studied aspect concerns the possibility of conferring self-responsive functions to polymeric matrices [3,13,19,20,21,22,23,24]. Among these smart abilities, there are self-healing [13], self-heating [3,22], and self-sensing functions [19,20,21,23,24]. Self-sensing polymeric systems have been designed using CNTs and graphene-based nanoparticles [19,20,21,23,24]. In particular, Hosseinzadeh et al. [19] used polyethylene terephthalate as a flexible substrate for the deposition of graphene and its derivatives. Among the investigated multi-layered structures, hydrogenated graphene oxide has been found to be characterized by a higher gauge factor with a value of 300.5. The responses of these systems did not show any hysteresis in cyclical measurements.

Herrera et al. [20] investigated the piezoresistive behavior of composites based on vinyl ester and multi-walled carbon nanotube applying a load in axial tension and compression mode. During load cycles, for the deformation value in the plastic regime, the electrical resistance does not return to its original value, indicating that this parameter is sensitive to plastic deformations. Similar results have been obtained by Vertuccio et al. [23,24]. Referring to Ref. [23], an epoxy resin with high mechanical performance (T_g_ = 260 °C) has been used as a hosting matrix. In this case, the study showed that the AC measurements are more effective than those in DC, due to the combined action of the resistances and capacitances that determine the overall electrical response of the material.

Based on these results, Vertuccio et al. demonstrated that a system based on epoxy resin and carbon nanotubes can be used as a strain-sensitive coating on aeronautical CFRP [24], where the gauge factor value of 4.7 corresponding to the coated CFRP is higher than those of many thermosetting resins based on carbon nanotubes.

A further improvement of the gauge factor was obtained by Spinelli et al. [21] when the carbonaceous particle introduced into the epoxy matrix was a 2D filler. In this last case, two different carbon-based fillers, multi-walled carbon nanotubes and exfoliated graphite characterized by a very different aspect ratio, were incorporated in the same insulating resin. The piezoresistive response of the resulting nanocomposites evidences different values of the gauge factor, 4 for the 1D system and 39 for the 2D system. The difference in the change in contact resistance among graphite layers and CNTs in the resin was hypothesized to be relevant in determining the sensitivity of the formulated bulk strain sensors.

The typical investigation techniques used to detect the water content in the material and the effects that follow are gravimetrical methods, dynamic mechanical, and size measurements carried out as a function of immersion time in the water [5]. The combined results of the different techniques allow the relating of the water uptake with the swelling phenomena and the plasticization phenomena. The gravimetric analysis defines the amount of the absorbed moisture, which is usually associated with mechanisms of moisture uptake, usually defined based on modeling approaches and verified by experimental data. Dynamic mechanical analysis provides information on plasticization phenomena and the cross-linking level [5,7,25]. Thickness measurements allow the correlation of the matrix structural changes with the mechanisms of water absorption by the study of the swelling phenomenon [26,27,28]. An approach by the use of electrical measurements, such as dielectric analysis, allows the investigation of the nature of the interaction of water with the matrix. More specifically, these measurements allowed the identification of the water in two states, as clustered droplets and bound to polar groups in the matrix [29]. When the water exists as in the first condition, the dielectric signature is similar to that of bulk water, while when the molecules of water become linked to the epoxy matrix, by hydrogen bonding, due to the presence of polar groups containing hydrogen and oxygen, the dielectric relaxation behavior is different [30]. Although a good correlation has been found between dielectric data and water uptake in the first stage of absorption up to a quasi-equilibrium state, a drop in the permittivity for long immersion times, and a subsequent trend of the permittivity to grow, simultaneously, an increasing water absorption has been found. It would seem that plasticization phenomena determine this fluctuating trend of dielectric permittivity [31,32,33].

In this work, epoxy/CNT nanocomposites were prepared with the aim to study two relevant aspects. The first was the monitoring of plasticization phenomena induced by water aging; the second was the identification of an easy methodology for detecting these phenomena. 

The evaluation of the resistive response of the nanocomposite loaded with 0.1% by weight of CNTs, detected through measurements of electrical resistance, has proven to be a very simple and effective way to achieve the prefixed goals. This is possible because the nanocomposite manifests a very interesting correlation between the changes in the electrical resistance and the plasticization phenomena due to the amount of absorbed water.

Gravimetric characterization of the system associated with an anomalous non-Fickian model allowed the evaluation of the kinetics of water absorption. The change in the electrical resistance of the nanocomposite retraces, in perfect agreement with the water absorption kinetics, allowed the correlating of the electrical measurement both with the water uptake and with the changes in the structural arrangement of the material.

## 2. Materials and Methods 

The components of the epoxy matrix are described in Table 1. The weight ratio between the epoxy precursor diglycidyl ether of bisphenol A (DGEBA) and the hardener 4,4 diaminodiphenylsulfone (DDS) was 10/2.85. Multi-walled carbon nanotubes (CNTs), 3100 Grade, were supplied from Nanocyl S.A. The morphological parameters of the CNTs have been described in a previously published paper [34]. The filler was dispersed in the precursor by the ultra-sonication process (Hielscher model UP200S-24 kHz) for 20 min. The obtained blend was mixed with the hardener by mechanical stirring at 120 °C for 1 h. A double step was used for the curing cycle. In particular, the mixtures were cured at 150 °C for 1 h followed by 3 h at 220 °C. The curing cycle used was chosen after thermal tests in which the absence of thermal degradation phenomena was verified (see Figures S1 and S2 of the Supplementary Materials). Differential scanning calorimetry investigation proved that the adopted curing cycle allows a curing degree value of 100% for the filled and unfilled resin (see Figure S3 of the Supplementary Materials). The analysis and characterization procedures are summarized in Table 2.

In particular, regarding the sorption-electrical test, the samples were conditioned under vacuum for 24 h, at a temperature of 120 °C, to ensure complete dryness, and afterward, they were placed into distilled water chambers maintained at a constant temperature of 30 °C. The integrity of the obtained samples was checked by SEM analysis (see Figure S4a of the Supplementary Materials), whose images show the absence of porosity on the surface of the samples. An etching treatment of the sample allowed removal of the epoxy matrix in the composite, exposing the carbon nanotubes and their arrangement within the matrix. The Figure S4b image (see Supplementary Materials) allows one to state that the distribution of the filler in the matrix is quite uniform. The electrical measurements were performed in such a way as to avoid any heating phenomenon due to the joule effect. The samples were weighed using a digital balance (resolution = 0.01 mg) to evaluate the amount percentage of the water uptake. The specimens were regularly removed, dried using clean tissue paper, in order to ensure the removal of excess surface water, and finally weighed. Subsequently, an electrical resistance measurement was carried out according to the standards described in Table 2 and, finally, the samples were placed again into the water bath. The water amount percentage (%) was determined from Equation (1)

(1) $$∆M/M_{0}=100\cdot \frac{M_{t}-M_{0}}{M_{0}}$$

where M_t_ and M_0_ are the weight of the specimen at time t and the initial weight of the dry specimen, respectively. The equilibrium concentration of water (M_∞_) is calculated considering the maximum amount of absorbed water after 610 h, when ∆M/M_0_ presents a plateau behavior.

## 3. Results and Discussion

### 3.1. Electrical, Mechanical, and Water Absorption Properties

#### 3.1.1. Electrical Properties

**Figure 1:**
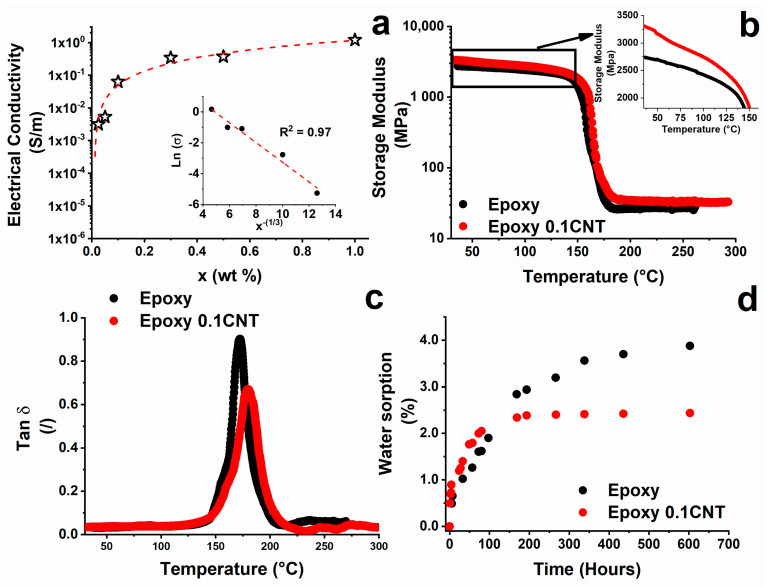
(**a**) Volume electrical conductivity vs. CNT weight percentage (inset: electrical conductivity versus x^−1/3^ for CNT weight percentage (x) above the EPT); (**b**) storage modulus; (**c**) tan δ of the unfilled epoxy and filled epoxy with 0.1% by weight of multi-walled carbon nanotubes; (**d**) water uptake as a function of the immersion time for unfilled and filled epoxy resin.

In the first stage of the experimental activities, physical characterization of the epoxy system with and without the filler was performed to choose the more performant sample for this kind of investigation. The concentration of 0.1%wt. of CNTs was proven to be suitable for the investigation. For this system, the correlation between electrical properties and decay in the mechanical properties, due to the water sorption, was studied. The system choice of the nanocomposite with 0.1% by weight of CNTs was made, taking into consideration the results of the electrical measurements. Figure 1a shows the trend of the electrical conductivity of the filled resin as a function of the filler percentage.

The percolation theory [35,36,37] explains the observed behavior. The electrical conductivity value (*σ*) jumps by several orders of magnitude compared to the value of the unfilled matrix. When this insulator-to-conductor transition is observed, a conductive network in the matrix allows the electrons to flow through the material. The conduction in CNT composites can be explained by considering that conductive paths, allowing the material to convert from an insulator to a conductor, are formed in the composite when the CNT concentration *(x*) increases over a threshold value *x_c_*. The percolation theory describes the dependence of the conductivity σ on the filler concentration by a scaling law of the form:

(2) $$\sigma =\sigma_{0}{\left(x-x_{c}\right)}^{t}$$

where *x_c_* is the electrical percolation threshold (EPT) and t is an exponent depending on the system dimensionality. In particular, Bauhofer [35] and Kovacs [37], based on a global survey of the data available in the literature, presented some general results concerning a systematic correlation of material characteristics (polymeric matrices, the CNT type, method of synthesis, processing, etc.) and parameters that describe the law of percolation. 

The inset of Figure 1a shows the electrical conductivity (in natural logarithmic scale) versus x^−1/3^ for concentrations (*x*) above the EPT. The obtained linear fitting allows the assertion that the tunneling effect is the electrical transport mechanism in the composite, in agreement with the traditional approach discussed in the literature [38,39,40]. In previous works [21,23,24,41,42], it has been found that a filled material that exhibits piezoresistive characteristics with respect to an external stimulus (i.e., tensile or flexural stress) exhibits greater sensitivity when the concentration of the filler approaches the EPT. In fact, the resistance variations obtained are much greater than those that occur when the content of the filler is well beyond the percolation threshold value. This is because, near the percolation threshold, the conductive network of the carbon nanotubes is characterized by larger tunneling distances between one contact and the other than those that would occur with high concentrations of fillers. Using the same approach, with the aim of maximizing the resistive characteristic of the system compared to an external stimulus, which, in this case, is due to phenomena of structural relaxation induced by water absorption, the concentration of 0.1% by weight of nanotubes was chosen. This value is next to the EPT (see Figure 1a), which, in our case, was found for values below 0.05% by weight of the filler.

#### 3.1.2. Mechanical and Water Absorption Properties

Figure 1b,c show the behavior of the storage modulus and the tan δ, respectively, of the filled and unfilled samples. For both systems, the glass transition temperature is in the temperature range between 130 °C and 220 °C (see Figure 1c), but a small shift in the temperature peak, which goes from 171 °C for the unfilled epoxy resin to 180 °C for the filled resin, is observed. A reinforcement effect, due to the nanofiller, is clearly deduced by the increase in the glass transition temperature (see Figure 1c) detected for the nanocomposite. Furthermore, a very slight reinforcement effect, due to the presence of the nanofiller, is also detected in the values of the storage modulus, both in the range below T_g_ (see inset in Figure 1b) and in the rubber phase (beyond T_g_), as it can be deduced by the position of the red curve, which is always upon the black curve (see Figure 1b). The improvement of the mechanical properties is the result of a network structure modified by the presence of the nanofiller and the interface between the CNT-polymer [43,44,45]. The good dispersion of the filler allows a reduction in liquid water sorption, as shown in Figure 1d. In the case of the nanocomposite, the maximum water sorption value is reduced from 3.9% to 2.4%, which is equivalent to a reduction of about 62.5%. This result importantly depends on the structure of the composite matrix, in which factors such as the nature and extension of interaction between the polymer and nanotubes, crosslink density, and mobility of polymer segments play a crucial role. Variations in the glass transition temperature are due to the balancing of opposite phenomena. Generally, for the same curing condition, the presence of the nanofiller in the polymeric matrix tends to decrease the curing degree with respect to the unfilled matrix. This effect is poorly reflected on the T_g_ value because the tendency for T_g_ to decrease is counterbalanced by the restricted mobility of the polymer chains in the vicinity of high-aspect-ratio carbon nanotubes [11,46]. These opposite effects result in an almost unchanged value of T_g_ for the matrix and the nanocomposite [11]. This consideration agrees well with the results of Figure 1c, where almost the same value of T_g_ is obtained for the unfilled resin and the nanocomposite.

The results obtained in Figure 1d can be explained if the nature of the crosslinking reactions is considered. The crosslinking reactions between the precursor and the amine hardener involve the opening of the epoxy ring by reaction with amine hydrogen or with the formed hydroxyl, with the formation of hydroxyl groups, as shown in Scheme S6 (see Supplementary Materials). The presence of the network of carbon nanotubes represents an obstacle to the formation of cross-linking points. From a chemical point of view, the polyaddition reactions that generate -OH groups are reduced. The -OH groups in the polymeric matrix can form hydrogen bonds with the water molecules by increasing the sorption water. The presence of carbon nanotubes, therefore, reduces the number of polar sites, and this causes a decrease in the amount of adsorbed water. This effect can be deduced by the consideration that, after a long time and all the plasticization effects, the water sorption for the nanocomposite still remains lower compared with the unfilled resin. FTIR measurements (see Figure S5 of the Supplementary Materials) demonstrated that the introduction of carbon nanotubes in the epoxy resin causes a reduction in the amount of hydroxyl groups of about 23%.

### 3.2. Thermomechanical and Water Diffusion Analysis (Water Aging Effect)

In order to define the structural changes undergone by the filled matrix due to the effect of water aging, DMA analysis was carried out under three different conditions of the sample: dry, wet at 1.2%, and 1.9% of water uptake.

**Figure 2:**
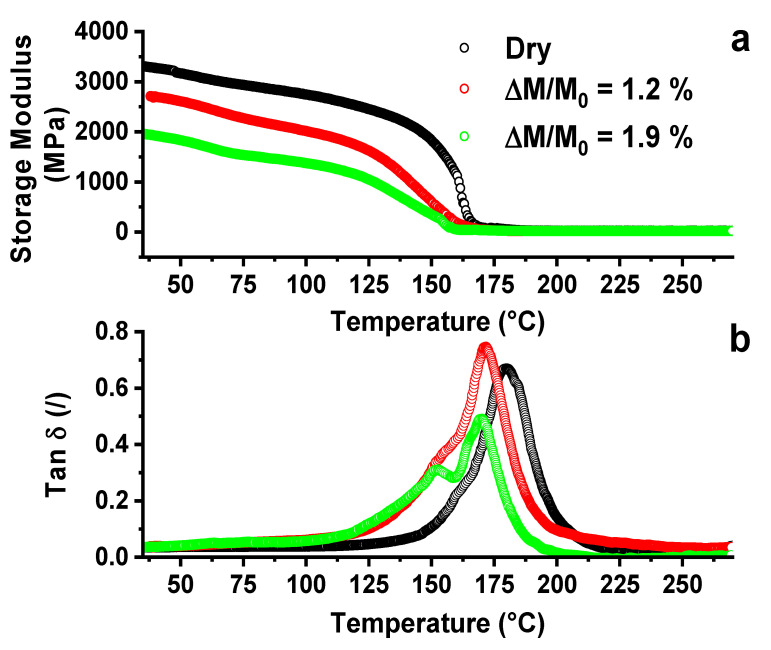
(**a**) Storage modulus and (**b**) tan δ of the filled epoxy with 0.1% by weight of CNTs at different ∆M/M_0_ values.

As expected, the water aging causes a progressive reduction in the mechanical properties with the increase in the water uptake. A reduction in storage modulus is found in the whole temperature range from 30 °C to 180 °C, with a consequent reduction in the value of the glass transition temperature. This behavior is typical of epoxy resins, composed of epoxy precursors bi- and tetra-functional, and DDS [5,6] (see Table 1). As previously described, after the curing reactions, the epoxy matrix presents polar sites due to the hydroxyl groups obtained from the crosslinking process [5,47,48]. The interaction of the water, which penetrates, creates hydrogen bonds with the polar sites, producing relaxation and plasticization phenomena in the matrix. Figure 2b shows a shift in the temperature value of the main peak, for the aged resin, toward lower temperatures. It is well known from polymer theory that the relaxation process in polymers, especially in composites, are complex processes, which stem from different mechanisms [49].

**Figure 3:**
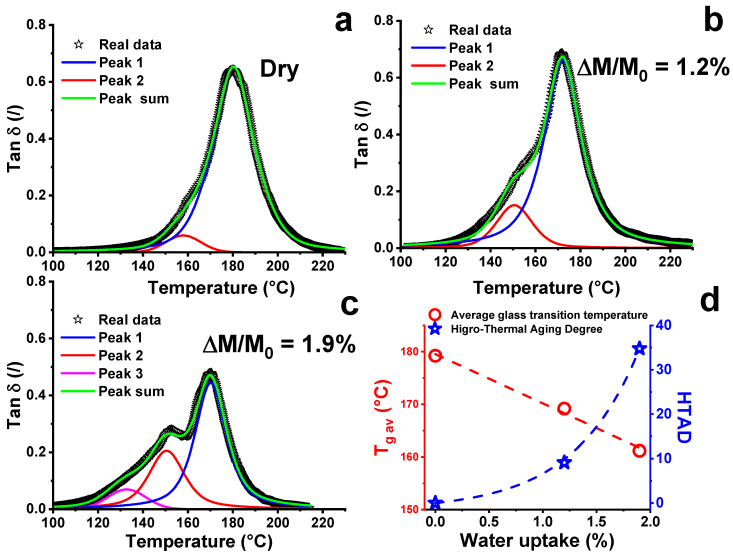
Master curve of the multiple α_i_-relaxations of the tan δ relating to the composite under different conditions: (**a**) dry; (**b**) water amount of 1.2%; (**c**) water amount of 1.9%; (**d**) average value of the glass transition temperature (T_g av_.) and Hydro-Thermal Aging Degree (HTAD) as a function of water amount.

Among various theoretical models that have been proposed to describe these complex effects, the assumption that the recorded process consists of discrete elementary relaxation processes constitutes a realistic and practical approach. These processes can be analyzed by the study of the glass transition temperature of the polymer. The glass transition process may be considered as being controlled by the intrinsic flexibility of the chain segments of the polymer, which depends on the free volume available within the polymer [49]. Conformational changes can only occur when there is sufficient free volume for the chain segment movements. The free volume is assumed to be present throughout the polymer. Moreover, according to the approach of the flexibility of the chain segments, the variation in chemical structure also involves an intermolecular contribution [49]. Thus, the glass transition can be influenced by both the intra- and intermolecular contributions. In order to analyze the described effect, a single glass transition temperature value is not adequate to describe the occurring events, because the glass transition temperature is not placed on a single value but covers a range of temperatures. Furthermore, the plasticization phenomena, due to the presence of water in the matrix, also cause enlargements in the shape of the tan δ peak, without determining relevant variations in the value of the temperature peak [11]. In any case, the variation in the shape of the tan δ peak causes a reduction in mechanical properties, such as the decrease in the values of the storage modulus (see Figure 3). All these considerations lead us to conclude that the different relaxation phenomena, causing changes in the tan δ profile, can be studied considering the peak of tan δ composed of multiple relaxation mechanisms, which are activated at different temperatures. Hence, tan δ can be assumed as a master curve of multiple α_i_-relaxations. The main peak, first, moves to lower temperature values from 180 °C to 171 °C, and then its height decreases from an initial value of 0.68 to a final value of 0.5 when the system contains an amount of water of 1.9%. This implies that the wet composite exhibits less elastic behavior than the dry composite does. The significant change in the width of the peak suggests a broader distribution of relaxation times, presumably also due to the nanoparticle–polymer interactions, which contribute to widening the temperature range corresponding to the activation of the molecular relaxation movements. In addition to this, for all systems, a shoulder below the main peak is observed. This shoulder is different both in intensity and in temperature value for the three investigated cases. This indicates the occurrence of a larger fraction of material involved in the relaxation phenomena, which are also activated at lower temperature with respect to the dry sample. The main mechanism (corresponding to the more intense peak) is caused by the reaction of the hardener agent with the epoxy groups of the precursor, giving rise to the main α-relaxation, while the less intense peak, on the left of the main peak, is the result of a different effect that is better analyzed considering the results of Figure 3.

Figure 3 shows the tan δ profile, approximated to different peaks according to the approach suggested by Stimoniaris et al. [49], where a broad α-relaxation peak results in a superposition of multiple relaxation mechanisms (α_i_), each with a characteristic transition temperature T_i_. The different peaks were calculated by applying a resolution algorithm based on the Levenberg–Marquardt method [50]. To reduce the number of adjustable parameters, the baseline and the peak equation was fixed. The peak function is a mixed Gauss–Lorentz equation [51]:

(3) $$f\left(x\right)=\left(1-L\right)Hexp\left[-4ln\left(2\right){\left(\frac{x-x_{0}}{w}\right)}^{2}\right]+LH{\left[{\left(\frac{x-x_{0}}{w}\right)}^{2}+1\right]}^{-1}$$

where *x*_0_ = the peak position; *H* = peak height; *w* = FWHH (Full-Width at Half-Height); *L* = fraction of Lorentz character.

The dry system (see Figure 3a) presents two peaks, the main one centered at about 180 °C and the second centered at the temperature of 158 °C. The presence of two peaks in the dry system indicates the presence of matrix fractions with different crosslinking density values [5]. This behavior is due to the nonstoichiometric precursor/hardener ratio used in the manufacturing of the samples. In fact, the amount of hardener is in defect with respect to the stoichiometric amount. The reduced amount of hardener causes the presence of chain segments that are more mobile, which coexist with structures that are more cross-linked, and determine a fraction at lower T_g_. The increase in the water uptake causes a reduction in both the position and the height of the main peaks, up to the wet sample at 1.9% water, which presents a third peak. The contribution of each reticulated structure to the formation of the overall behavior of the material depends on the characteristic weight of the different peaks; for this reason, in order to quantify the deviation from the structure of the dry matrix, the average values of the glass transition temperature and the network homogeneity index (IH)) are introduced [49]. In particular, the average value of the glass transition temperature is expressed by the following equation:

(4) $$T_{g\;\mathrm{av}.}=\frac{\sum \text{​}T_{\mathrm{peak}\;i}*A_{i}}{\sum \text{​}A_{i}}$$

where T_peak i_ is the temperature and A_i_ is the area of the i-peak, while the network homogeneity (IH) is expressed by the following equation:

(5) $$\mathrm{IH}=\frac{A_{1}}{A_{1}+\sum \text{​}A_{i}}$$

where A_1_ is the bounded area of the main peak and IH represents the deviation from a ‘perfect’ network, which is expected to possess an α-relaxation peak having a symmetrical bell-shaped distribution.

In light of the parameters considered, a percentage change in network homogeneity (IH) compared to that obtained in the dry system was evaluated considering a new term of comparison defined as Hydro-Thermal Aging Degree (HTAD):

(6) $$\mathrm{HTAD}=100*\left(1-\frac{{\mathrm{IH}}_{\mathrm{wet}}}{{\mathrm{IH}}_{\mathrm{dry}}}\right)$$

where IH_dry_ and IH_wet_ are the homogeneity network under the dry condition and wet condition, respectively. These parameter values are summarized in Table 3.

**Figure 4:**
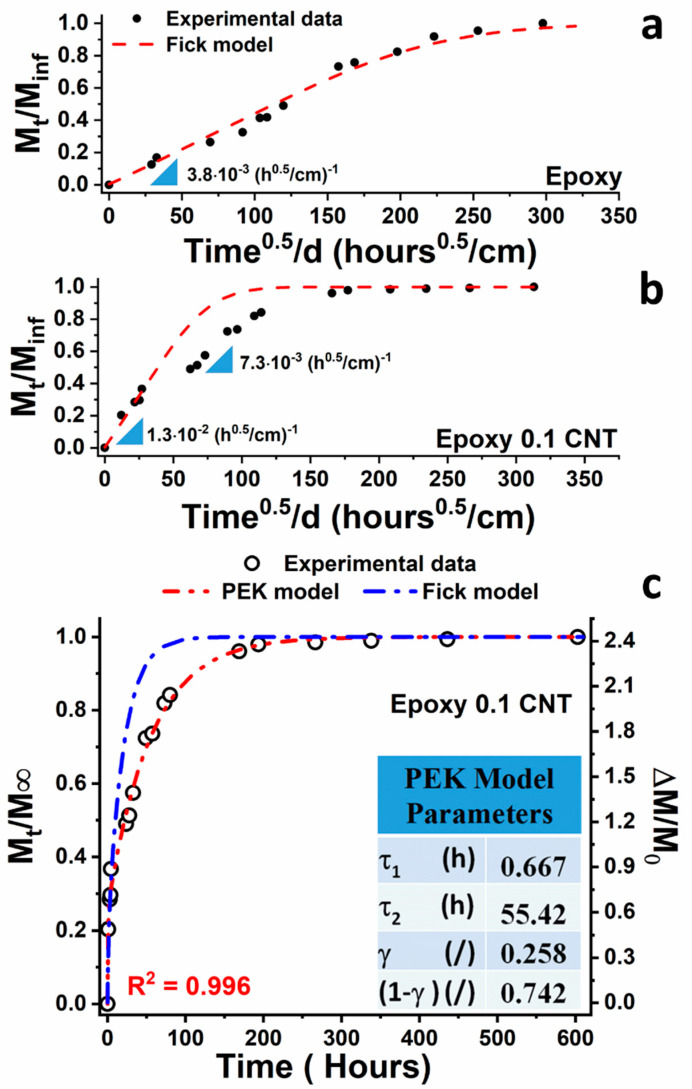
M_t_/M_∞_ against the square root of time of (**a**) the pristine resin and (**b**) resin with 0.1% CNT. (**c**) Water absorption curves as a function of the immersion time ((M_t_/M_∞_) left vertical axis; ∆M/M_0_ right vertical axis); symbols are experimental data; curves are calculated by the Fick diffusion (blue) and PEK models (red).

The values of the parameters, shown in Table 3, indicate that the temperature relative to the main peak registers a shift of 10 °C with a water uptake of about 1% and that this value remains substantially unchanged for subsequent absorption up to a value of about 2%. As it can be clearly seen from Figure 3 and Figure 4, the temperature of the main peak does not allow one to identify the aging state of the sample. The previously exposed alternative, in which a glass transition temperature with an average value is evaluated, in accordance with Equation (4), defines the real water aging state of the sample. Contrary to the linear decrease in the T_g av_ with the absorbed water (10 °C/1% water uptake), an exponential behavior of the HTAD is found. In particular, the HTAD grows by about 10% for a water uptake value of 1.2%, and then it reaches a value of 35% for a double value of water uptake. This trend seems to presage that the mechanisms of water penetration into the matrix are not univocal and that the structural reorganization of the polymer, caused by the water aging, greatly influences diffusion phenomena. In this regard, an investigation about the kinetics of water absorption was carried out.

In Figure 4a, the reduced curve, M_t_/M_∞_, as a function of the square root of time, normalized for the thickness d (cm) of the pristine resin, is reported, whereas in Figure 4b, the reduced curve of the filled system is shown. In the sample, shown in Figure 4a, a Fickian behavior (see Equation (7)) can be observed, that is, a linear dependence of the reduced sorption on the square root of time, up to a value of 0.8, beyond which a curvature is detected, which represents the equilibrium of sorption. In the case of the CNT-based epoxy resin, the first part, extending up to M_t_/M_∞_ = 0.4, is steeper than the previous curve of Figure 4a, giving a higher angular coefficient. However, a second stage appears after the value M_t_/M_∞_ of 0.4, slowing down the diffusion process. It is evident that the diffusion of water molecules into the composite sample with carbon nanotubes follows two trends, in which the first part follows the Fick model, whereas a second step exhibits anomalous behavior where the diffusion process is slower. Although it is not possible to identify a unique water sorption ratio for the filled system, Figure 4b show that the diffusion phenomena are slower for the raw resin. Probably, on the one hand, the introduction of carbon nanotubes reduces the polar sites, as previously described and supported in the Supplementary Materials. On the other hand, the presence of a second phase, reasonably correspondent with the polymer forming a layer around the carbon nanotubes, results in a greater free volume, which allows a faster water permeation.

Several models have been proposed for modeling the water diffusion behavior in polymers. The most common model in which water absorption is considered to be independent of water concentration obeys Fick’s law [52]:

(7) $$\frac{M_{t}}{M_{\infty }}=1-\overset{\infty }{\underset{0}{\sum }}\frac{8}{{\left(2n+1\right)}^{2}\pi^{2}}exp\left[-\frac{D{\left(2n+1\right)}^{2}\pi^{2}t}{l^{2}}\right]$$

where *M_t_* and *M_∞_* are the water uptake at time *t* and the maximum water uptake, respectively; *D* is diffusivity; and *l* is the sample thickness. The solution for Fick’s law for short times (*M_t_*/*M*_∞_ < 0.6) then reduces to the following equation for the initial stage of diffusion:

(8) $$\frac{M_{t}}{M_{\infty }}=\frac{4}{l}{\left(\frac{Dt}{\pi }\right)}^{\frac{1}{2}}$$

In our case, Fick’s model greatly deviates from the experimental data trend (see Figure 4). Good correspondence is obtained only for water uptake values corresponding to M_t_/M_∞_ less than 0.3 for which a value of the diffusion coefficient *D* = 8.3 × 10^−9^ cm^2^/s has been calculated. It should be noted that in Fick’s law, the water transport is controlled by diffusion, while the contribution due to the molecular relaxation processes is considered negligible. Although the water uptake of the epoxy matrixes can generally be described by Fick’s law [53,54], in some glassy polymers, diffusion of water may follow anomalous or ‘‘non-Fickian’’ behavior [8,55,56]. In our case, the best fitting was obtained with an approach based on the viscoelastic nature of polymers and on relaxation phenomena, accelerated by the water penetration in the matrix, which are expressed in the plasticization and aging effects [7,11,57]. In particular, the time-varying diffusivity model (DTVD) was used. If a DTVD model is considered, the water sorption of epoxy resin, which undergoes plasticization phenomena, can be modeled by parallel independent first-order processes, using the parallel exponential kinetics (PEK) model [58,59]. More specifically, the PEK model can be viewed as a simplified case of what could be a potentially infinite assembly of series-coupled Kelvin–Voigt viscoelastic elements [60], where the constant coefficient of diffusion is replaced by a decreasing function of time (by analogy with a relaxation modulus for a viscoelastic solid). The experimental data are well approximated by two kinetic steps (R^2^ = 0.996) as follows:

(9) $$\frac{M_{t}}{M_{\infty }}=\gamma *\left[1-\exp\left(\frac{-t}{\tau_{1}}\right)\right]+\left(1-\gamma \right)*\left[1-\exp\left(\frac{-t}{\tau_{2}}\right)\right]$$

where $\tau_{\iota }$, and *γ* and (1 − *γ*) correspond to the time constants and the fraction of water uptake for which the individual processes occur, respectively. The values obtained from the best fitting are shown in Figure 4c. The first of the two kinetic processes is fast while the second is slower, as evidenced by the rate constant values *t*_1_ and *t*_2_. The fast and slow sorption steps are related to different Higro Thermal Aging Degree values; in fact, when the HTAD is below 10%, the *M_t_*/M_inf_ behavior is almost linear with the aging time until the water uptake achieves the value of 1%. After this last value, a strong change in slope of the *M_t_*/M_inf_ occurs. In this second step, the variation rate of HTAD is higher than the previous one (see blue curve of Figure 3), making the kinetic diffusion process slower. This double step trend is also found in the variation in electrical resistance as a function of the water aging time, as shown in the next section.

### 3.3. Resistive Analysis 

**Figure 5:**
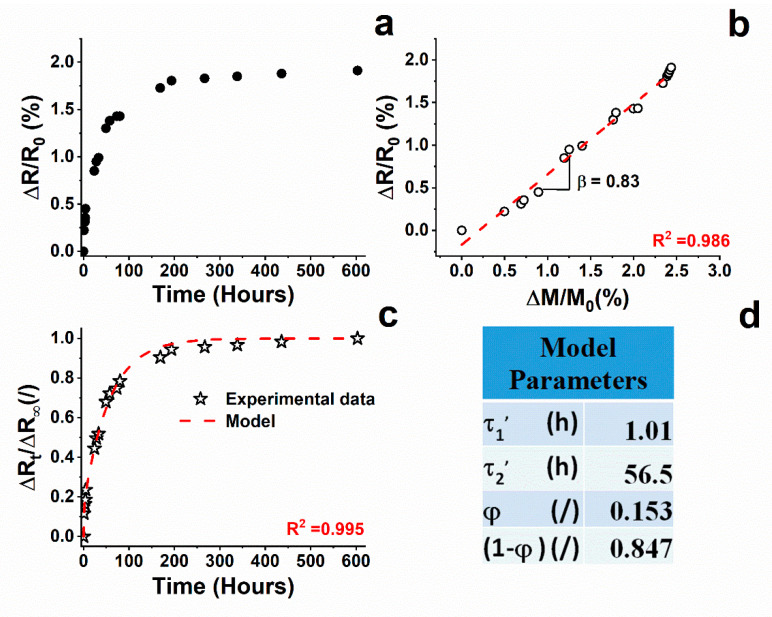
(**a**) Resistance change ratio (∆R/R_0_) as a function of the immersion time; (**b**) resistance change ratio (∆R/R_0_) vs. water amount percentage (symbols are experimental data, red line is calculated by linear fitting); (**c**) R_t_/R_∞_ curves as a function of the immersion time (symbols are experimental data, red curve is calculated by fitting Equation (10)); (**d**) parameter values of Equation (10).

Figure 5a shows the electrical resistance change ratio versus the water aging for filled resin. The variation in time of the resistance change ratio is similar to that of water absorption. In fact, it presents a first linear section with a high slope in the first 30 h of the test, a second step in which there is a decrease in the slope due to the reduction in the sorption rate, and a final step in which there is no variation in electrical resistance by setting it as a plateau value. The resistance plateau value corresponds to the value of the maximum absorbed water amount by the material (M_∞_). The water sorption-resistive behavior of the composite is closely related to the electrical resistance of the sample; one way to quantify the sensitivity of the system to water uptake variations is to plot ΔR/R_0_ vs. ΔM/M_0_, as shown in Figure 5b. In this case, a linear trend (R^2^= 0.986) is found with a slope (β) of around 0.8. Generally, polymers filled with electrically conductive particles can have piezoresistive characteristics, whose sensitivity to mechanical external stimulus, as stress or strain, is expressed with a factor defined as the gauge factor [21,61]. Similarly, in our case, it is possible to define a sensitivity factor (β) to the water uptake and then to the aging of the material due to the water sorption, which allows the association of the electrical resistance measurement to the amount of the absorbed water. The good correspondence between the change in the electrical resistance and the water uptake can be explained thanks to the phenomenon of swelling. This phenomenon is related to the increase in the size of the sample caused by water absorption. The swelling induces deformations, which affect the mechanisms of water absorption and related structural reorganizations in the polymer [27]. In previous studies, the kinetics behavior of the swelling is found to be similar to that of water absorption in the cases in which the kinetic water sorption process can be identified by two stages [11,27,55].

In the presence of electrically conductive fillers, such as carbon nanotubes, the swelling phenomenon can affect the contact resistance in different ways. The most obvious is an increase in interparticle contact distance that leads to an increase in tunneling resistance. In any case, structural variations induced by the swelling phenomenon cause a modification of the inter-particle contacts [62]. Following a double exponential decay model used to fit the swelling [26] and water absorption [63,64], the ratio R − R_0_/R_∞_ − R_0_ (R_t_/R_∞_) can be expressed by the following equation:

(10) $$\frac{R_{t}}{R_{\infty }}=\varphi *\left[1-\exp\left(\frac{-t}{\tau_{1}^{\prime }}\right)\right]+\left(1-\varphi \right)*\left[1-\exp\left(\frac{-t}{\tau_{2}^{\prime }}\right)\right]$$

where φ corresponds to the quasi-equilibrium point of the two-stage kinetic model, and τ’_1_ and τ’_2_ are time constants. The values of τ’_1_ and τ’_2_ (see Figure 5d) obtained by Equation (10) are very close to those of τ_1_ and τ_2_ calculated with Equation (9). This confirms that the water diffusion process is based on continuous relaxation phenomena due to plasticization effects. The difference in the value between φ and γ is attributable to the correspondence between the amount of water absorbed and the resulting swelling obtained. The obtained results highlight that the resistive response of the composite can be used to monitor the amount of the water uptake and the changes in the structural arrangement of the material subjected to aging in a humid environment.

## 4. Conclusions

The relationship between electrical resistance and water uptake was explored on a carbon nanotubes-based epoxy resin. The water uptake behavior followed a non-Fickian trend where the swelling phenomena affected the mechanisms of water absorption and related structural reorganizations in the polymer. The plasticization effects of epoxy were detected by dynamic mechanical analysis and electrical measurement. The behavior of the electrical resistance was similar to that of water absorption both modeled by a parallel exponential kinetics approach. The increasing interparticle contact distance, due to the swelling phenomena, led to an increase in tunneling resistance and a variation in the bulk electrical resistance of the composite system. The resistive response of the composite can be used to monitor the changes in the structure of the material subjected to water aging.

## Figures and Tables

**Table 1:** Material components.

Product	Formula	Supplier	Functional Group
**DGEBA**	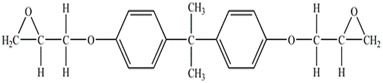	Sigma Aldrich	**2**
**DDS**	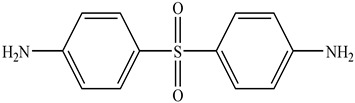	Sigma Aldrich	2 *

* 4 active hydrogen atoms.

**Table 2:** Characterization methods.

**DMA**	**Specific**
Sample dimension	4 × 10 × 35 mm^3^
Configuration	Dual Cantilever
Displacement amplitude	0.001
Frequency operating condition	1 Hz
Temperature operating condition	from 30 °C to 300 °C
Scanning rate	3 °C/min^−1^
**Electrical measurement**	**Specific**
Sample dimension	0.5 × 10 × 10 mm^3^
Configuration	Cabot Test Method (CTM) E043 based on ASTM D4496
Contact (gold)	coating deposition by Agar Auto Sputter Coater
**Water sorption test**	**Specific**
Sample dimension	0.5 × 10 × 10 mm^3^
Temperature	30 °C
Environmental condition	Liquid water
**Image of the Epoxy 0.1CNT sample**	
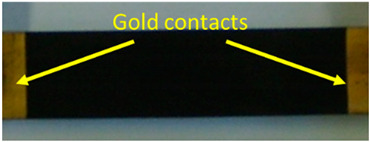	

**Table 3:** Computational analysis results for the filled system at different water amount percentages.

Water Sorption (%)	T_g main peak_ (°C)	T_g av_ (°C)	IH (/)	HTAD (%)
0	180.1	179.2	0.95	0.00
1.2	171.7	169.2	0.86	9.12
1.9	170.1	161.2	0.62	34.78

## Data Availability

Data are contained within the article and Supplementary Materials.

## Supplementary Materials

The following are available online at https://www.mdpi.com/article/10.3390/nano11092183/s1, Figure S1: DSC heating curve of the epoxy resin, Figure S2: Thermal analysis of the sample: (a) DSC heating curve of the uncured resin; (b) TGA heating curve in air of the cured resin, Figure S3: DSC curves of (a) the uncured and (b) cured epoxy resins, Figure S4: Scanning electron microscope images of sample Epoxy 0.1% by weight of carbon nanotubes (a) without oxidative treatment and (b) after etching treatment, Figure S5: FTIR spectra of (a) the components of epoxy (precursor and hardener) and (b) cured epoxy, Figure S6: Scheme of cross-linking reactions between epoxy pre-polymers and amine hardeners, Figure S7: FTIR spectra of (a) epoxy and (b) epoxy with 0.1% by weight of carbon nanotubes. References [65,66,67] are cited in the supplementary materials.
