# Supplementary material for: Resistive Response of Carbon Nanotube-Based Composites Subjected to Water Aging
Source: Nanomaterials (Basel). 2021 Aug 25;11(9):2183. doi: 10.3390/nano11092183 (PMC8465806; doi:10.3390/nano11092183)
Supplement: Supplementary file 1 [file nanomaterials-11-02183-s001.zip › nanomaterials-1314725-supplementary.pdf]

nanomaterials

Supplementary Materials

Resistive Response of Carbon Nanotube-Based Composites Subjected to Water Aging

Liberata Guadagno and Luigi Vertuccio *

Department of Industrial Engineering, University of Salerno, Via Giovanni Paolo II, 84084 Fisciano, SA, Italy; lguadagno@unisa.it
* Correspondence: lvertuccio@unisa.it

1. Thermal Analysis

Differential Scanning Calorimetry (DSC) was carried out on the samples under investigation using a thermal analyzer Mettler DSC 822/400 (Mettler-Toledo, Columbus, OH, USA) equipped with DSC cell purged with nitrogen and chilled with liquid nitrogen for sub-ambient measurements. The selected temperature range was 30–450 °C at a heating rate of 10 °C/min. Thermogravimetric analysis (TGA) was carried out in air on samples using a Mettler TGA/SDTA 851 thermal analyzer (Mettler-Toledo, Columbus, OH, USA). Temperature range was 25–900 °C at a heating rate of 10 °C/min.

A differential scanning calorimeter was used for the dynamic curing experiments under a nitrogen flow. To select suitable temperatures for the required isothermal experiments in oven, a dynamic DSC scan at a heating rate of 10 °C/min was first obtained (see Figure S1). Values of temperature above, but near the onset of curing reactions, were chosen. This method avoids the improper choice of temperature, which may be too high or too low. The curing temperature value of 220 °C is in the optimal interval of temperature for a curing cycle. For this reason, the curing cycle for all systems is 150 °C for 1 h followed by 3 h at 220 °C.

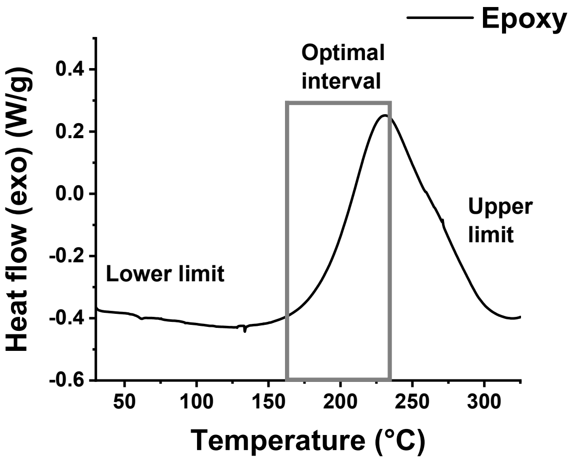

Figure S1. DSC heating curve of the epoxy resin.

The choice of a curing temperature of the 220 °C was made after preliminary experiments, in which it was ascertained that no oxidation phenomena occur for this value of temperature. Figure S2a shows a dynamic curing cycle by DSC in which the test was carried out up to 450 °C and Figure S2b shows the TGA curve of the sample in the same range of temperatures. The temperature corresponding to the degradation phenomena is clearly visible from the TGA curve. The comparison between Figure S2a and Figure S2b highlights that the initial oxidation phenomena occur only beyond the temperature of 330 °C.

In particular, the first step of weight loss (about 65%) is detected in the temperature range of 330 °C–450 °C, and therefore in a temperature range beyond that selected to polymerize the polymer.

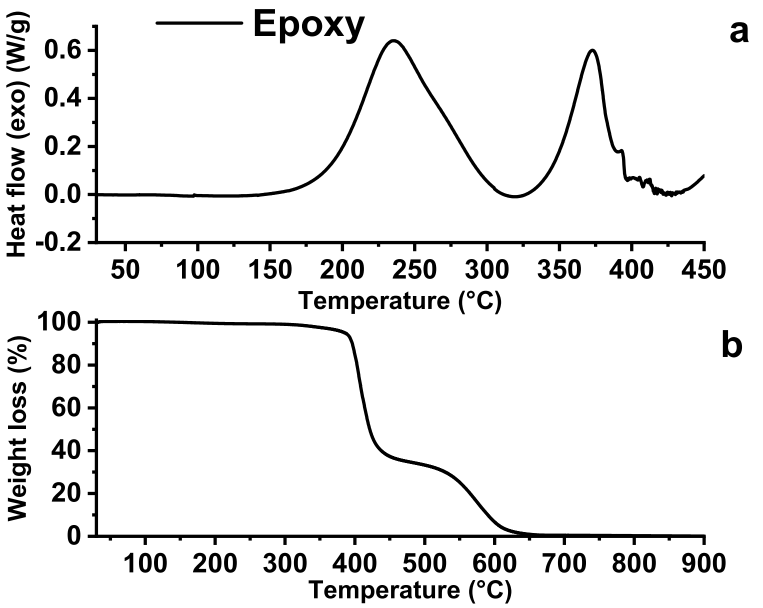

Figure S2. Thermal analysis of the sample: (**a**) DSC heating curve of the uncured resin; (**b**) TGA heating curve in air of the cured resin.

It is worth noting that the understanding of the effect of the nanofiller on the mechanical spectrum is complicated by the overlapping of several factors. For investigating the influence of the nanofiller on the dynamic mechanical properties of the analyzed formulations, the curing degree [1] plays a crucial role because it affects the glass transition temperature. DSC has been used for the estimation of the curing degree of the samples under the assumption that the exothermic heat evolved during cure is proportional to the extent of reaction. This assumption has already been adopted by other authors [2,3]. The curing degree (CD) can be determined from the total heat of reaction (ΔH_{tot}) of the curing reactions and the residual heat of reaction ($\Delta H_{residual}$) of the partially cured epoxy resin as follows:

$$DC = 100 \times \frac{\Delta H_{tot} - \Delta H_{residual}}{\Delta H_T} \quad (1)$$

Dynamic runs were made after the curing cycle (150 °C for 1 h followed by 3 h at 220 °C) to obtain the ($\Delta H_{residual}$). ΔH_{tot} and $\Delta H_{residual}$ are the areas under dynamic thermograms, respectively.

Considering that, the nanofilled sample has been cured using the same curing cycle, the presence of the filler, in the amount of 0.1% by weight, not affects the curing degree. From the lack of the $\Delta H_{residual}$ in the second run DSC, it results that, unfilled and filled sample respectively, manifest a curing degree value of 100% (see Figure S3).

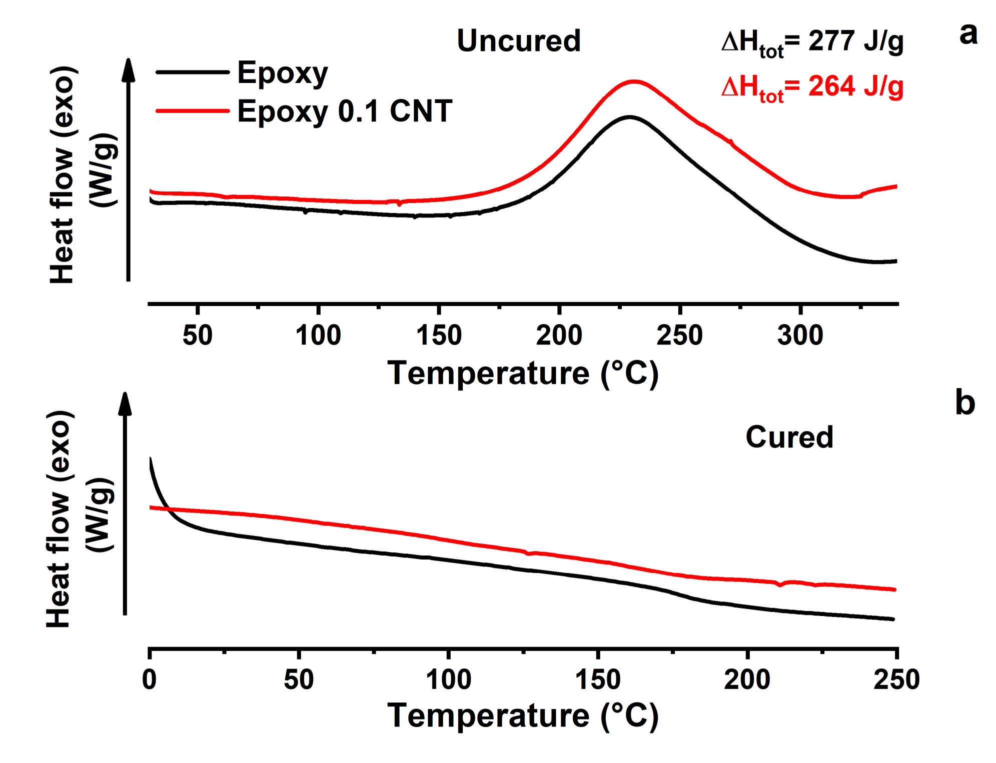

Figure S3. DSC curves of: (**a**) the uncured and (**b**) cured epoxy resins.

2. Morphological Analysis

"High resolution scanning electron microscope (HRSEM) micrographs were obtained with a field emission SEM apparatus (JSM-6700F, JEOL, Aksishima, Japan) instrument operating at 3 kV. The absence of porosity in the obtained samples was checked by morphological analysis. The SEM image (see Figure S4a) represents the sample containing the carbon nanotubes at a concentration of 0.1% by weight. The Figure shows the untreated system (not etched-without any type of oxidative treatment). The surface of the sample is uniform and without the presence of pores. In addition, magnifications of smaller areas show the absence of pores and the presence of carbon nanotubes.

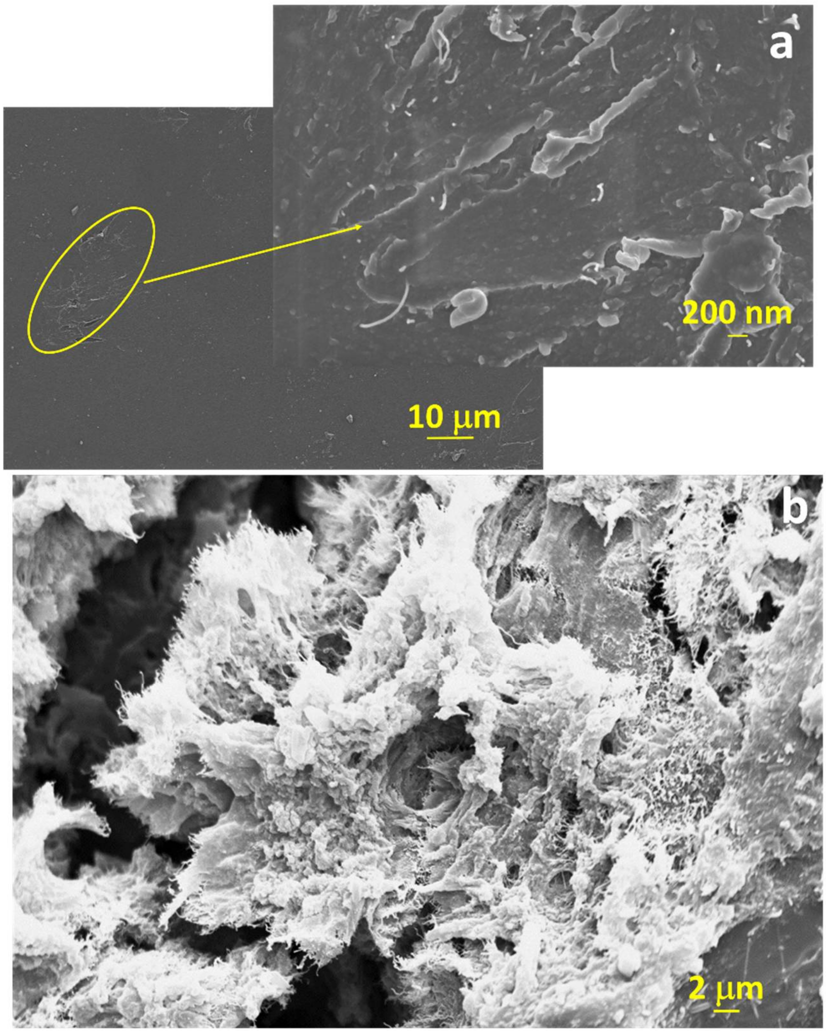

Figure S4. Scanning Electron Microscopes image of sample Epoxy 0.1% by weight of carbon nanotubes (**a**) without oxidative treatment; (**b**) after etching treatment.

Figure S4b shows the SEM images of the composites where an etching reactant has been employed to partially remove the epoxy resin to explore more accurately the arrangement of the fillers within the matrix. The etching reagent was prepared by stirring 1.0 g potassium permanganate in a solution mixture of 95 mL sulfuric acid (95–97%) and 48 mL orthophosphoric acid (85%). The filled resins were immersed into the fresh etching reagent at room temperature and held under agitation for 24 h. Subsequent washings were done using a cold mixture of two parts by volume of concentrated sulfuric acid and seven parts of water. Afterward the samples were washed again with 30% aqueous hydrogen peroxide to remove any manganese dioxide. The samples were finally washed with distilled water and kept under vacuum for 2 days at 80 °C before being subjected to morphological analysis. The image allows to state that the distribution of the filler in the matrix is uniform. In the empty areas, corresponding to the areas where the matrix has been removed, it is possible to glimpse the electrical conductive junctions due to the presence of carbon nanotubes not far from each other.

3. IR Characterization

FTIR spectra were obtained at a resolution of 2.0 cm^{-1} with a FTIR (BRUKER Vertex70, Bruker Optics Inc., Billerica, MA, USA) spectrometer equipped with deuterated triglycine sulfate detector and a KBr beam splitter, using KBr pellets. The frequency scale was internally calibrated to 0.01 cm^{-1} using a He-Ne laser. 32 signal scans were averaged

to reduce the noise. In order to check the reduction of the -OH group in the epoxy matrix for effect of introduction of the filler in the matrix, IR measurements were carried out.

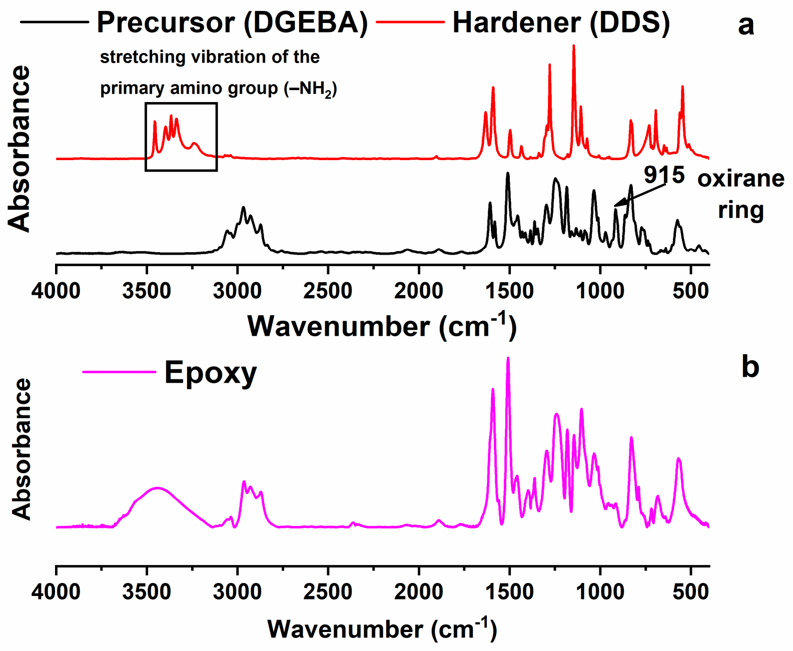

Figure S5. FTIR spectra of (**a**) the components of epoxy (Precursor and Hardener); (**b**) cured epoxy.

Figure S5a shows the FTIR spectrum of the precursor and the hardener before the curing cycle, while Figure S5b shows the FTIR spectrum of the epoxy resin after the curing cycle. The main peaks have been identified in Figure S5a and assigned either to the precursor and to hardener respectively. In particular, the main signal of the oxirane ring is at 915 cm^{-1} (in the epoxy precursor) whereas the peaks 3369 cm^{-1}, 3298 cm^{-1}, and 3174 cm^{-1}, are due to stretching vibration of the primary amino group ($–NH_2$) in the hardener . The crosslinking reactions between precursor and amine hardener involve the opening of the epoxy ring by reaction with amine hydrogen or with the formed hydroxyl, with the formation of hydroxyl groups, as shown in the scheme S6 and checked in the FTIR spectrum in Figure S5b, in the wavenumber range from 3150 cm^{-1} to 3670 cm^{-1}. The occurred reaction is checked by the reduction of the peak to 915 cm^{-1} and by the formation of the hydroxyl group in the in wavenumber range from 3150 cm^{-1} to 3670 cm^{-1} (see Figure S5b).

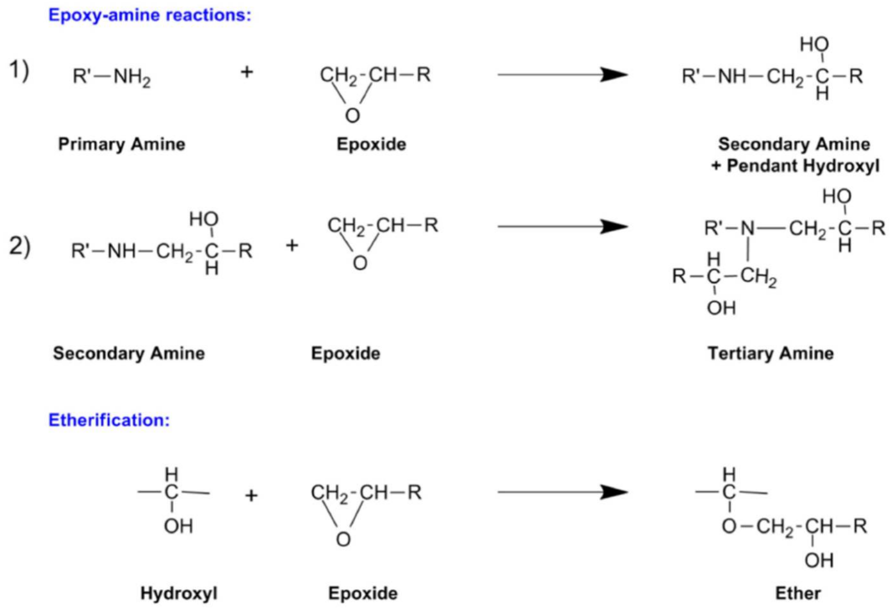

Figure S6. Scheme of cross-linking reactions between epoxy pre-polymers and amine hardeners.

Figure S7 shows the FTIR spectra of: a) Epoxy; b) Epoxy with 0.1% by weight of carbon nanotubes.

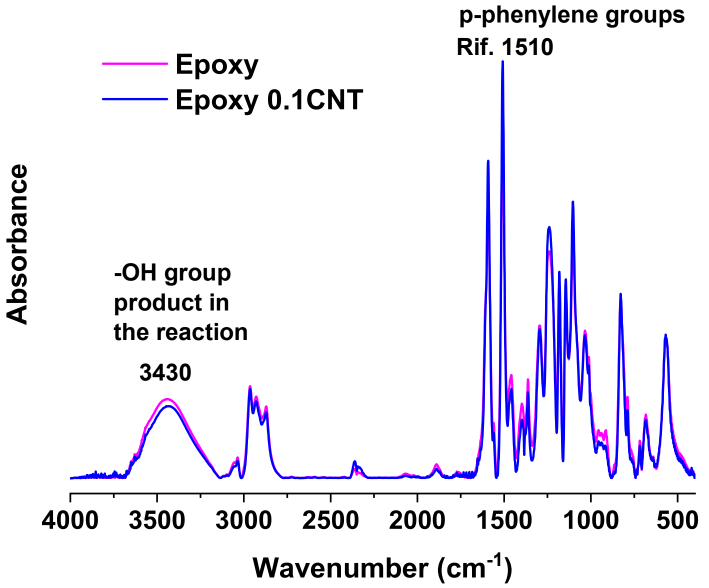

Figure S7. FTIR spectra of Epoxy and Epoxy with 0.1% by weight of carbon nanotubes.

The inclusion of filler in the resin causes a decrease in the intensity of the -OH peak. In order to evaluate the effective reduction of the peak relative to the hydroxyl group, the intensity of this group has been normalized to the peak at 1510 cm^{-1} associated with the p-phenylene groups, which are assumed chemically unmodified during the reaction. This type of normalization has proven to be very effective also for other systems [4]. The ratio, $R = A_{\text{-OHpeak}}/A_{\text{peak1510}}$, of the subtended areas is evaluated for Epoxy systems and filled epoxy system respectively. Figure S7 shows in detail the peaks taken into account for this

investigation. The ratio R varies of about 23% from a value of 3.21 for the epoxy resin to a value of 2.48 for the filled resin.

References

1. Guadagno, L.; Raimondo, M.; Vittoria, V.; Vertuccio, L.; Naddeo, C.; Russo, S.; De Vivo, B.; Lamberti, P.; Spinelli, G.; Tucci, V. Development of epoxy mixtures for application in aeronautics and aerospace. *Rsc Adv.* **2014**, *4*, 15474–15488.
2. Edwards, G.; Ng, Q. Elution behavior of model compounds in gel permeation chromatography. J. *Polym. Sci. Polym. Symp*. **1968**, *21*, 105–117.
3. Horie, K.; Hiura, H.; Sawada, M.; Mita, I.; Kambe, H. Calorimetric investigation of polymerization reactions. III. Curing reaction of epoxides with amines. *J. Polym. Sci. A Polym. Chem.* **1970**, *8*, 1357–1372.
4. Karayannidou, E.G.; Achilias, D.S.; Sideridou, I.D. Cure kinetics of epoxy–amine resins used in the restoration of works of art from glass or ceramic. *Eur. Polym. J.* **2006**, *42*, 3311–3323.
